# Magnetic-Assisted Cell Alignment within a Magnetic Nanoparticle-Decorated Reduced Graphene Oxide/Collagen 3D Nanocomposite Hydrogel

**DOI:** 10.3390/nano9091293

**Published:** 2019-09-10

**Authors:** Mallesh Santhosh, Jin-Ha Choi, Jeong-Woo Choi

**Affiliations:** 1 Center for Integrated Biotechnology, Sogang University, 35 Baekbeom-ro (Sinsu-dong), Mapo-gu, Seoul 121-742, Korea; 2 Department of Chemical and Biomolecular Engineering, Sogang University, 35 Baekbeom-ro (Sinsu-dong), Mapo-gu, Seoul 121-742, Korea

**Keywords:** collagen hydrogel, 3D scaffold, reduced graphene oxide, magnetic nanoparticles, aligned cells

## Abstract

Hydrogel scaffolds are particularly interesting for applications in tissue engineering because of their ability to create a favorable environment which mimics in vivo conditions. However, the hierarchically ordered anisotropic structure which is found in many native tissues and cellular components is hard to achieve in 3D scaffolds. In this work, we report the incorporation of magnetic nanoparticle-decorated reduced graphene oxide (m-rGO) within a collagen hydrogel. This magneto-responsive m-rGO aligned within the collagen hydrogel during gelation with the application of a low external magnetic field. This nanocomposite hydrogel with magnetically aligned m-rGO flakes is capable of encapsulating neuroblastoma cells (SH-SY5Y), promoting cell differentiation and inducing oriented cell growth owing to its excellent biocompatibility and electrical conductivity. The directionally oriented and differentiated SH-SY5Y cells within the m-rGO collagen hydrogel showed propagation of calcium signal along the direction of orientation. This method can be applied to creating magnetically responsive materials with potential for various biomedical applications.

## 1. Introduction

The development of functional three-dimensional (3D) scaffolds with appropriate biological, physicochemical, and mechanical characteristics that allow to mimic the in vivo conditions of natural extracellular matrix (ECM) is required in tissue engineering [1]. A hierarchically ordered anisotropic structure is one of the common features of biological tissues. In many complex tissues (e.g., muscles and nerves), alignment is critical to provide the tissue with proper structure and mechanical properties, which direct its functions in many instances [2]. The development of functional, aligned engineered tissue constructs would be potentially beneficial for the treatment of conditions which involve the central nervous system (CNS), such as neurodegenerative diseases (e.g., Parkinson’s disease and Alzheimer disease) and traumatic brain injuries [3].

Because of its important, many approaches have been developed to achieve anisotropy in engineered tissues [4,5], most of which are limited to a planar surface, while some were aimed at developing 3D scaffold. Various strategies such as patterned surfaces [6], aligned fibrils generated via electrospinning [7,8], strain induction [9], and microfluidics [10] have been developed to provide cell-directing cues. Recently, methods to develop engineered 3D scaffold with unidirectional orientation have gained more importance. Several strategies have been introduced to confer anisotropy to 3D constructs, which in many cases utilize techniques such as freeze-drying [11,12], shear flow [13], short-pulse lasers [14], and particulate leaching and strong external forces, such as electric forces [15], magnetic fields [16], and mechanical stress [17] to align fibers anisotropically.

Various biochemical cues (such as retinoic acid, valproic acid) [18,19] and biophysical cues [20] (nanotopographical features) play a major role in inducing neuronal differentiation of neuronal stem cells (NSCs). Additionally, various conductive biocompatible materials have also been reported to induce neuronal differentiation [21,22]. Graphene and its derivatives are extensively used in the field of biomedical engineering owing to their excellent electrical conductivity, mechanical strength, and favorable biocompatibility [6,23]. These materials have been proved to be effective in enhancing neuronal differentiation of NSCs and promoting neurite outgrowth. In fact, graphene was found to enhance the differentiation of human neural stem cells (hNSCs) into neurons rather than glial cells, which is highly desired. Moreover, graphene-based materials have been used to monitor the differentiation of neuronal cells [24,25,26,27]. Graphene oxide (GO) and its reduced form (rGO) are characterized by the presence of a variable amount of oxygen-containing functional groups (mainly hydroxyls and epoxies) in the lattice of sp^2^-bonded carbon atoms [28,29,30]. These groups enhance their hydrophilicity and adsorptive properties and, hence, have excellent biocompatibility. These derivatives of graphene were also found to enhance the neuronal differentiation of NSCs and SH-SY5Y cells [31,32].

Especially for the development of 3D engineered neuronal tissue scaffolds, it is highly desired to recreate an aligned 3D ECM architecture with biocompatible, conductive nanocomposite materials (such as graphene and its derivatives) which not only assist in improving the mechanical properties but also act as cues for cellular alignment and enhance the differentiation of neurons. 

Recently, a composite hydrogel of Matrigel [33] and collagen [34] with polydispersed magnetically aligned chains of spherical iron oxide particles has been described for creating aligned 3D matrices. In these nanocomposite hydrogel scaffolds, aligned magnetic nanoparticles provide a supporting structure for the orientation of the cells. Alternatively, to reduce the concentration of iron oxide nanoparticles required for the alignment, which may limit their biomedical application, researcher have developed a dual hydrogel system called anisogel, composed of a microgel loaded with a low dose of superoxide paramagnetic iron oxide nanoparticles (SPIONs) that orient in mT magnetic fields before being fixed in a cross-linked hydrogel [35,36]. Although these systems require a very low concentration of SPIONs and are particularly good in promoting oriented neuronal cell growth, the use of materials that promote enhanced neuronal differentiation additionally to alignment is more useful in neuronal tissue engineering.

To address these issues, we developed a collagen hydrogel-based 3D nanocomposite scaffold, containing magnetic nanoparticle-decorated reduced-graphene oxide (m-rGO) nanosheets with the aim of developing an aligned 3D scaffold which favors neuronal differentiation and directs the orientated growth of the cells. Collagen is an attractive biomolecule ubiquitously found in a broad range of tissues and has excellent biocompatibility and dynamic structural manipulation characteristics. On the other hand, rGO has excellent biocompatibility and conductivity and can enhance neuronal differentiation. Magnetic-responsive m-rGO nanosheets were prepared by attaching SPIONs over graphene oxide sheets with the assistance of a multi-domain protein, bovine serum albumin (BSA), which dispersed inside the liquid collagen suspension and allowed its solidification in the presence of a low magnetic field (~50 mT). Then, m-rGO within the gel positioned along the magnetic field, inducing collagen gel fibers orientation. When neuroblastoma cells SH-SY5Y were incorporated within this nanocomposite 3D scaffold, aligned m-rGO and collagen fibers acted as spatial guidance cues to induce unidirectional growth of the cells and promote their differentiation into neurons. The differentiated neurons inside this nanocomposite hydrogel showed unidirectional calcium signal propagation. 

## 2. Materials and Methods 

### 2.1. Materials

Collagen (type-I, rat tail extract, in 0.02 M acetic acid, initial concentration of 3.35–4 mg·mL^−1^) was procured from BD-Biosciences (Bedford, MA, USA). Graphene oxide (1 mg·mL^−1^) was obtained from graphene supermarket. BSA, hydrazine mono hydrate, 5 nm iron oxide nanoparticles were purchased from Sigma-Aldrich (St. Louis, MO, USA). Hoechst, Alexa Fluor™ 546 Phalloidin, Calcein Am, and propidium iodide (PI) were purchased from Thermo Fisher Scientific (MA, USA). Rabbit anti-tyrosine hydroxylase (TH) and Texas red- and fluorescein-conjugated secondary antibodies were procured from Abcam (Cambridge, UK). Oregon GreenTM 488 BAPTA and pluronic acid were procured from life technologies (MA, USA).

### 2.2. Synthesis of M-rGO

Magnetically responsive reduced-graphene oxide (m-rGO) nanosheets were synthesized through the decoration of graphene oxide nanosheets with 5 nm SPIONs by following a reported method [37] with slight modifications. Briefly, 2 mL of GO stock solution was mixed with 200 µL of 0.5% of BSA and 25 µL of 5% SPIONs, and the suspension was stirred for 1 h to allow physical adsorption of SPIONs over the GO flakes which was assisted by the interaction with BSA. The solution was kept at 80 °C with continuous stirring. After 1 h, 10 µL of hydrazine was added and allowed to react for 20 h under constant stirring. After the completion of reaction, the mixture was allowed to cool and centrifuged at 12,000 rpm for 15 min. The pellets were washed twice in distilled water and dispersed in distilled water through sonication. After that, the m-rGO solution was stored at 4 °C.

### 2.3. Preparation of Collagen and M-rGO/Collagen Composites

A collagen stock solution was neutralized, and its final concentration was brought to 3 mg·mL^−1^ by the addition of 15 µL 10× phosphate buffered saline (PBS) and 2 µL of 1 N NaOH to 100 µL of collagen solution in 1.5 mL microtubes. To a freshly prepared collagen solution (100 µL), 30 µL of m-rGO was added, and the solution was diluted with 170 µL of Dulbecco’s modified Eagle medium DMEM media to achieve the final concentrations of 1 mg·mL^−1^ and 0.1 mg·mL^−1^ of collagen and m-rGO, respectively. For the preparation of cell-seeded hydrogels, 170 µL of DMEM containing SH-SY5Y neuroblastoma cells to achieve a final cell density of 2 × 10^6^ in the hydrogel solution was slowly mixed with the nanocomposite hydrogel, avoiding the formation of air bubbles. These steps were performed on ice to avoid undesired gelation. Then, 50 µL of the cell-seeded nanocomposite hydrogel was placed into squared polydimethylsiloxane (PDMS) molds (1 cm × 1 cm). Each mold was placed in a 3D printed stage which transversely housed two magnets for magnetic actuation. The set up was placed in a 37 °C incubator for 1 h for gelation to occur. After gelation, the construct was transferred to a culture dish with growth media.

### 2.4. Cell Culture

The neuroblastoma cell line (SH-SY5Y) was obtained from ATCC (Manassas, VA, USA). DMEM supplemented with 1% antibiotics (streptomycin and penicillin) and 10% heat-inactivated fetal bovine serum (FBS) was used as the growth media. The cells were cultured at 37 °C in a humidified atmosphere in the presence of 5% CO_2_. The cells were grown in tissue culture-grade Petri dishes, and every 48 h of incubation, the medium was replaced with fresh medium; the cells were passaged when 80–90% confluence was reached. For cell differentiation, nanocomposite hydrogel constructs with SH-SY5Y cells were cultured in growth medium. After 24 h, the growth medium was replaced with a differentiation media DMEM supplemented with 1% FBS and 1 × 10^−5^ M retinoic acid (RA) to induce the neuronal differentiation of SH-SY5Y cells in 8 days of culture. The medium was replaced every alternative day.

### 2.5. Live/Dead Cell Assay

Cells viability within the nanocomposite hydrogels was assessed using calcein-AM (which stains live cells) and PI (which stains dead cells) staining. After 2, 5, and 7 days of culture, the hydrogels with encapsulated SH-SY5Y cells were washed with PBS and then incubated with calcein-AM (0.5 µg·mL^−1^) and 2 µg PI for 30 min at 37 °C. After that, the samples were washed with PBS to reduce the background fluorescence and visualized using confocal microscopy. Cell viability was evaluated by counting live and dead cells from four representative maximum intensity projections of the confocal images for each condition.

### 2.6. Immunofluorescence Staining 

Composite collagen gel constructs were rinsed with PBS, followed by fixation with 4% paraformaldehyde for 30 min at room temperature. The cultures were again washed with PBS and permeabilized with 0.5% Triton X-100 in PBS for 10 min. The gels were then incubated in a blocking solution containing 1% BSA in PBS. Then, the constructs were incubated with rabbit anti-TH (1:500) primary antibodies diluted in blocking solution for 3 h at room temperature. The samples were washed twice with PBS and then incubated with secondary antibodies for 1 h at room temperature. Finally, the cell nuclei were stained by using 2 µg·mL^−1^ of Hoechst in PBS. Images of the immuno-stained SH-SY5Y inside the 3D composite hydrogels were acquired using a laser scanning confocal microscope (LSM 710 Carl Zeiss) equipped with solid-state lasers (405, 488, 555, and 639 nm) with a 20× or 10× objective. Post-image processing such as maximum intensity projection, orthogonal view was performed using ZEN2 blue edition 2012 software (Carl Zeiss microscopy, GmbH, Germany).

### 2.7. Measurement of Cell Orientation 

After culturing for 5 d, the 3D constructs were fixed with 4% paraformaldehyde for 30 min and then washed with 1× PBS for three times. Then, they were permeabilized with 0.1% Triton X100 for 15 min at room temperature and washed three times with 1× PBS to remove Triton. The cells were incubated in Alexa Fluor 546 phalloidin (1:40 dilution in 1× PBS) and Hoechst (1:1000 dilution in 1× PBS) for 40 min at room temperature. Images of the cell morphology were acquired by confocal microscopy (LSM 710). Maximum intensity projection of the confocal images was used to analyze the orientation of the cells within the scaffolds. The orientation of SH-SY5Y was analyzed using OrientatioJ plug-in of ImageJ software (http://sites.imagej.net/BIG-EPFL/).

### 2.8. Calcium Imaging

The 3D constructs at 7 days were loaded with the membrane-permeable calcium-sensitive dye Oregon GreenTM 488 BAPTA (5 µM) and a pluronic acid 20% solution in DMSO at a ratio of 1:1 in 10 mM HEPES buffer containing 145 mM NaCl, 3 mM KCl, 1.5 mM CaCl_2_, 1 mM MgCl_2_, 10 mM glucose. After 1 h of incubation at 37 °C, the constructs were washed with HEPES buffer and kept at 37 °C for an additional half hour for dye esterification. Following this, the constructs were transferred to the microscopic stage for imaging. Time-lapse calcium images were acquired with LSM 710 by using a 40× water immersion objective with a spatial resolution of 128 × 128 pixels for 500 frames at a sample rate of 5 Hz. The image sequences were preprocessed by using the open-source image-processing package Fiji (http://fiji.sc/Fiji).

## 3. Results

### 3.1. Synthesis and Characterization of M-rGO

**Scheme 1:**
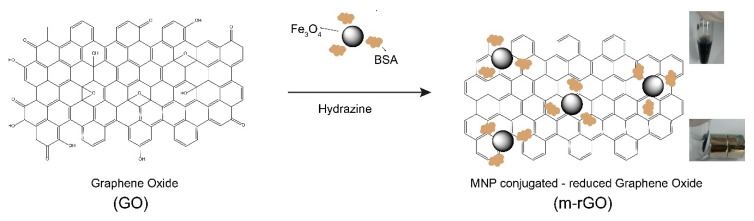
Scheme of the synthesis of magneto-responsive magnetic nanoparticle (MNP)-decorated reduced graphene oxide (m-rGO) via bovine serum albumin (BSA)-assisted decoration of superoxide paramagnetic iron oxide nanoparticles (SPIONs) on the surface of graphene oxide (GO).

**Figure 1:**
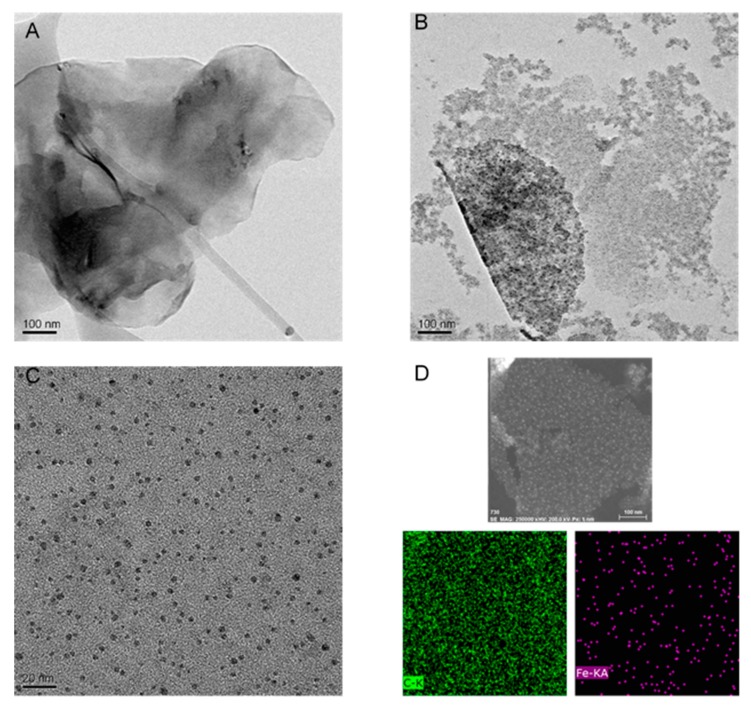
Transmission electron microscopic (TEM) images of GO (**A**) and m-rGO nano sheets (**B**). (**C**) TEM images of zoomed m-rGO with 5 nm diameter iron oxide nanoparticles (Scale bar, 20 nm). (**D**) Energy-dispersive X-ray spectroscopy (EDS) element mapping of a portion of m-rGO.

The GO sheets were reduced in situ and functionalized with magnetic nanoparticles with the assistance of the multi-domain protein BSA, which helped to maintain the magnetic nanoparticles absorbed on the surface of the GO sheets. The inherent reducing properties of BSA helped in the partial reduction of GO, which was further completely reduced to rGO by treatment with hydrazine at 80 °C (Scheme 1). The surface characterization of the GO sheets (before and after decoration with the magnetic nanoparticle) was assessed by transmission electron microscopy (TEM). The results indicated the presence of layers of graphene oxide (Figure 1A). The smooth and GO planar sheets clearly demonstrated the presence of a high surface area. TEM images of magnetic nanoparticle-decorated rGO sheets are shown in Figure 1B,C. Very evenly distributed magnetic nanoparticles with diameters of the order of a few nanometers (~5 nm) were observed over rGO sheets in the TEM images (Figure 1C). This clearly indicated the presence of strong interactions between rGO and the magnetic nanoparticles. The energy-dispersive X-ray spectroscopy (EDS) element mapping data also showed the good distribution of Fe (red) on the surface of rGO sheets (Figure 1D). Field emission scanning electron microscope (FESEM) images and EDS map spectra of GO and m-rGO are shown in Supplementary Figure S1 and Figure S2, respectively. Elemental composition analysis of m-rGO reflected the presence of Fe (~23%), while control GO showed no presence of Fe. These results further condirmed that the synthesized m-rGO nanosheets were coated with magnetic nanoparticles. Raman spectra of GO and m-rGO are shown in Figure S3. The D band (~1354 cm^−1^) corresponds to the disorder in the sp^2^ carbon network, and the G band (~1607 cm^−1^) is associated with the tangential vibrations of the sp^2^ carbon atoms in the hexagonal planes [38]. The intensity ratio of the D and G bands was employed to calculate the structural disorder; this ratio of the peaks increased during the reduction of GO to rGO, indicating an increase in the presence of sp^2^ carbon and, thus, GO reduction.

### 3.2. Biocompatibility of M-rGO/Collagen Scaffolds

**Figure 2:**
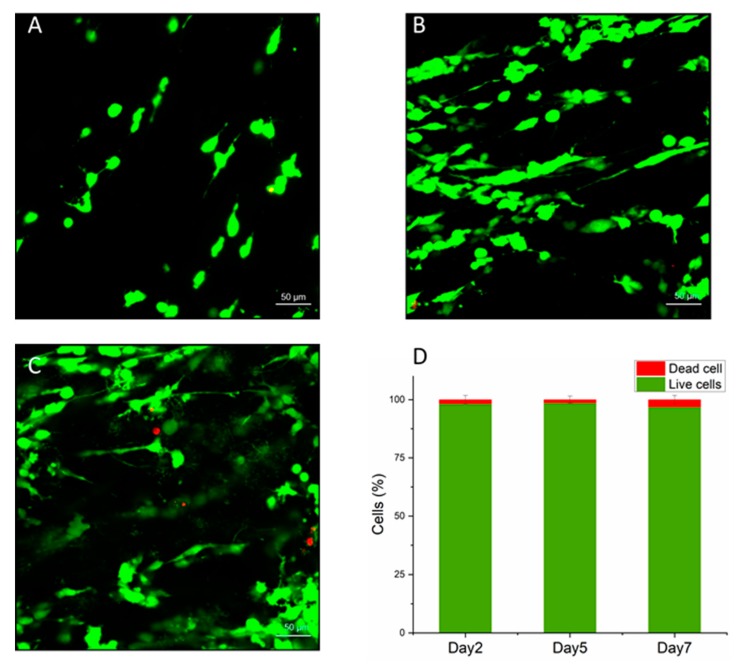
Viability and proliferation of SH-SY5Y cells in the m-rGO/collagen hydrogel scaffold. (**A**) Representative confocal fluorescence images of live and dead SH-SY5Y cells in the construct for 2 days (**A**), 5 days (**B**), and 7 days (**C**). (**D**) Quantification of cell viability.

To assess the viability of neuroblastoma cells within the m-rGO/collagen nanocomposite, we carried out a live/dead cell viability assay with calcein-AM and propidium iodide (PI). We observed that the viability of SH-SY5Y cells within the nanocomposite hydrogel was unaffected (Figure 2A–C). Most of the cells within the nanocomposite scaffolds remained alive and continued to proliferate within the scaffolds for over 7 days. The quantification results of the live/dead cell assay (Figure 2D) further confirmed that more than 95% of the cells remained viable within the 3D nanocomposite hydrogel throughout the experiments. The viability of SH-SY5Y cells at different concentrations of m-rGO was also assessed (Figure S4). The SH-SY5Y cells were found to be viable within the m-rGO/collagen composite hydrogel at an m-rGO concentration of 0.1 and 0.2 mg·mL^−1^ (Figure S4). These results indicate the excellent biocompatibility of the m-rGO/collagen composite hydrogel.

### 3.3. Orientation of Cells within M-rGO/Collagen Scaffolds

**Figure 3:**
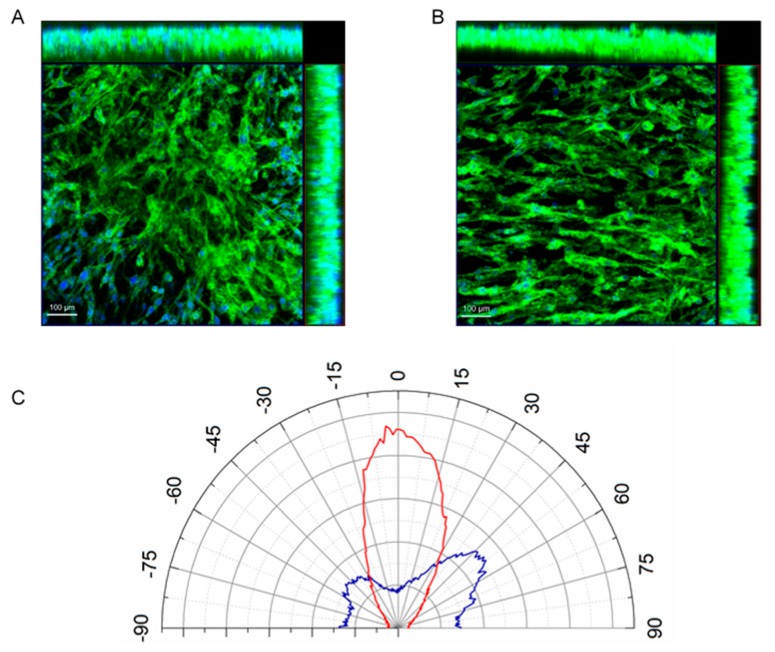
Morphology of SH-SY5Y cells in a random collagen hydrogel scaffold (**A**) and in an aligned m-rGO/collagen composite hydrogel (**B**). (**C**) Angular distribution of randomly oriented (blue trace) and aligned SH-SY5Y cells (red trace).

The application of a low magnetic field (~50 mT) during the process of gelation of the m-rGO/ collagen nanocomposite hydrogel resulted in the orientation of m-rGO along the direction of the field, which in turn assisted the directional gelation of the collagen fibers. The alignment of the collagen fibres was characterized by scanning electon microscope (SEM). The SEM images of control and aligned collagen are provided in Figure S5. The low- and high-magnification SEM images clearly show the oriented collagen fibres in an aligned collagen scaffold (Figure S5B,D) in contrast to a random collagen scaffold (Figure S5A,C). The effect of the m-rGO/collagen scaffold on cell morphology and directional growth was studied by incorporating SH-SY5Y cells inside collagen and its composite hydrogels. The cells were stained for F-actin by using phalloidin 546, and cell morphologies were studied using a confocal microscope. In the case of collagen hydrogels without m-rGO, lacking spatial guidance due to random collagen fiber orientation, we observed multi-directional growth of the cells, with F-actin filament stretched in all directions (Figure 3A). In contrast, in the case of m-rGO/collagen composite hydrogels, SH-SY5Y cells displayed unidirectional cell growth, and the neurites were elongated in the direction of the magnetic field (Figure 3B). The angular distribution of SH-SY5Y cells in 3D collagen and m-rGO/collagen is shown in Figure 3C. Within the m-rGO/collagen composite hydrogels, SH-SY5Y cells angular distribution showed a much narrower peaksas compared to SH-SY5Y cells within collagen hydrogels lacking m-rGO.

### 3.4. Differentiation of SH-SY5Y Cells within M-rGO/Collagen Scaffolds

**Figure 4:**
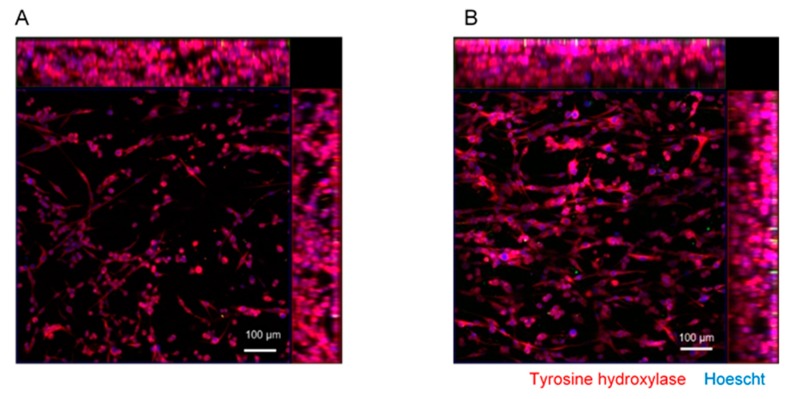
SH-SY5Y cell differentiation on m-rGO/collagen hydrogel scaffolds. Representative confocal immunofluorescence images of random (**A**) and aligned (**B**) collagen scaffolds. The cells were stained for the mature neuronal marker tyrosine hydroxylase (red); nuclei were stained with Hoechst (blue).

Studies were conducted to investigate the role of the incorporated m-rGO nanocomposite within the hydrogel scaffold in the differentiation of SH-SY5Y cells. We observed the formation of elongated neurites and the expression of mature neuronal markers such as TH, confirming the differentiation of SH-SY5Y cells (Figure 4). We carried out control experiments with magnetic nanoparticles (MNPs) without graphene oxide to evaluate the role of graphene oxide in neuronal differentiation. The cells within MNP/collagen nanocomposite scaffolds were also aligned (Figure S6B) with the magnetic field because of the collagen fibre orientation along the direction of the magnetic field [34]. The average neurite lengths were 143.90 ± 45.2 μm and 181.05 ± 35.8 µm for cells within the MNP/Collagen and the m-rGO nanocomposite scaffold, respectively. These reults demonstrate the capability of m-rGO to promote neurite growth in SH-SY5Y cells. 

**Figure 5:**
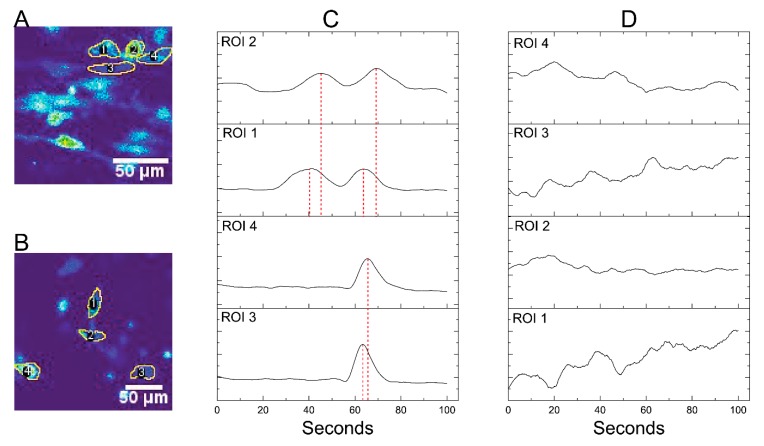
Spontaneous activity of the differentiated SH-SY5Y cells within the hydrogel scaffolds. Time-lapse image sequences (**A**) and (**B**) of measured calcium signals in a neuronal cell inside the composite hydrogels corresponding to supplementary movie S1 (aligned) and movie S2 (random), respectively. Normalized calcium transients of aligned (**C**) and randomly oriented cells (**D**).

We further studied the functionality of the differentiated neurons inside the nanocomposite hydrogels. We measured the calcium transients through the calcium indicator dye Oregon Green BAPTA. The initial time-lapse calcium videos of the constructs were processed by ImageJ software. Neurons were localized, and appropriate region of interest (ROIs) were selected. The differentiated SH-SY5Y cells within the nanocomposite hydrogelse exhibited spontaneous neuronal activity (Supplementary Video S1 and S2). Quantification of the signals in the ROIs of aligned (Figure 5A and Video S1) and un-aligned cells (Figure 5B and Video S2) was performed inside the hydrogel (Figure 5C,D respectively). In the case of aligned SH-SY5Y cells, calcium signal propagated along the direction of cellular orientation. Signal frequency quantification at different ROIs (1, 2, 3, and 4) showed a time lag between ROIs 1 and 2 as well as between ROIs 3 and 4 (red-dotted trace in Figure 5C). In contrast, in the case of un-aligned SH-SY5Y cells, calcium signal propagation was random (Figure 5D). These results indicate that in the case of aligned SH-SY5Y cells, calcium signals propagated along the direction of orientation.

## 4. Discussion

In this contribution, we have developed a collagen-based novel nanocomposite hydrogel scaffold containing magneto-responsive m-rGO nanosheets to fabricate a novel heterogeneous composite 3D scaffold for guiding SH-SY5Y cells’ oriented growth and mature neuronal differentiation. SH-SY5Y human neuroblastoma cells have some characters of NSCs such as neuronal differential capacity [39,40] and, hence, have been used extensively as an in vitro model for neurological studies, including neuronal differentiation and functions related to neurodegenerative processes and neurotoxicity [41,42]. It is still a great challenge to construct aligned 3D scaffold in situ to recapitulate in vivo conditions. Only a few methods have been devised so far for inducing cell alignment in 3D systems. Some methods use high magnetic fields (˃5 T) to align diamagnetic protein fibers inside a construct. Few methods use magnetic nanoparticles embedded inside a hydrogel (collagen, matrigel, alginate) to construct magneto-responsive tissue-engineered constructs. The concentration of MNPs in the order 1.5 mM reduces the viability of sensitive cells and is not desirable for in vivo applications [43]. Most recently, researchers have focused on developing a new type of dual hydrogel systems containing short magneto-responsive hydrogel fibers decorated with SPIONs which align inside the surrounding hydrogel upon the application of a low magnetic field (100 mT) [35]. The use of short fibres and low concentrations of SPIONs enhances the applicability of this method for in vivo studies. However, when developing neuronal tissue constructs, it would be more beneficial if the scaffolds are electroactive so that they can induce the orientated growth of nerve cells and promote the differentiation and network formation of NSCs. Most of the conductive materials lack biocompatibility [44]. However, the composite scaffolds containing graphene-based materials hold great promises in tissue engineering because of their excellent conductivity and biocompatibility. 

In the present study, we decorated the surface of graphene oxide nanosheets with SPIONs with the assistance of BSA, which maintained the magnetic nanoparticles adsorbed on the GO surface. The physical adsorption of BSA generates a predefined molecular coating that settled the saturation limit to the number of adsorbed molecules and provided more available sites for the adsorption of SPIONs [35]. These m-rGO flakes were incorporated within a collagen solution to create a composite hydrogel scaffold. The synthesized m-rGO/collagen scaffold has excellent biocompatibility and alignement topographical cues and can mimic the native ECM of the nerve tissue, successfully promote cell adhesion and proliferation, and induce the cells to grow directionally. The cell differentiation results showed that our m-rGO/collagen composite scaffolds can not only induce the differentiated SH-SY5Y neurites to arrange along the fibers’ direction, but also promote the maturation of SH-SY5Y cells, as indicated by the significant expression of the neuronal marker tyrosine hydroxylase. The differentiated neurons inside the composite hydrogel showed spontaneous electrical activity, with calcium signals propagating along the direction of cell orientation.

## 5. Conclusions

In this study, we designed and fabricated new composite hydrogel scaffolds composed of aligned magneto-responsive m-rGO within a collagen hydrogel scaffold, which exhibits excellent conductivity and biocompatibility. These heterogeneous composite scaffolds can effectively induce the oriented growth of SH-SY5Y cells and enhance SH-SY5Y cells neuronal differentiation and formation of neural networks. This work provides a simple method to fabricate aligned biocompatible 3D hydrogel scaffolds for controlling the differentiation and axon orientation of neuronal cells, which hold great potentials for creating magnetically responsive materials with potential for various biomedical applications.

## Supplementary Materials

The following are available online at https://www.mdpi.com/2079-4991/9/9/1293/s1, Figure S1: FESEM and EDS analysis of GO; Figure S2: FESEM and EDS analysis of m-rGO nanocomposite; Figure S3: Raman spectra of GO and m-rGo; Figure S4: Quantification of live/dead cells at different concentrations of m-rGO; Figure S5: Scanning electron microscope images of randomly oriented (A and C) and aligned collagen fibers (B and D); Figure S6: Cell morphology of SH-SY5Y cells within random (A and B) and aligned scaffolds (C and D) of MNP/collagen and m-rGO/collagen, respectively. (E) Average neurite length of SH-SY5Y cells; Video S1: Calcium image propagation in aligned SH-SY5Y cells within the 3D construct; Video S2: Calcium image propagation in un-aligned SH-SY5Y cells within the 3D construct.
