# Supplementary material for: Magnetic-Assisted Cell Alignment within a Magnetic Nanoparticle-Decorated Reduced Graphene Oxide/Collagen 3D Nanocomposite Hydrogel
Source: Nanomaterials (Basel). 2019 Sep 10;9(9):1293. doi: 10.3390/nano9091293 (PMC6781054; doi:10.3390/nano9091293)
Supplement: Supplementary file 1 [file nanomaterials-09-01293-s001.pdf]

nanomaterials

MDPI

Article

Magnetic-Assisted Cell Alignment within a Magnetic Nanoparticle-Decorated Reduced Graphene Oxide/ Collagen 3D Nanocomposite Hydrogel

Mallesh Santhosh [1,†], Jin-Ha Choi [2,†] and Jeong-Woo Choi [1,2,*]

[1] Center for Integrated Biotechnology, Sogang University, 35 Baekbeom-ro (Sinsu-dong), Mapo-gu, Seoul 121-742, Korea; santhosh@sogang.ac.kr

[2] Department of Chemical and Biomolecular Engineering, Sogang University, 35 Baekbeom-ro (Sinsu-dong), Mapo-gu, Seoul 121-742, Korea; jinhachoi@sogang.ac.kr

* Correspondence: jwchoi@sogang.ac.kr; Tel.: (+82) 2-705-8480; Fax: (+82) 2-718-1976

† These authors contributed equally to this work.

Received: 23 July 2019; Accepted: 8 September 2019; Published: 10 September 2019

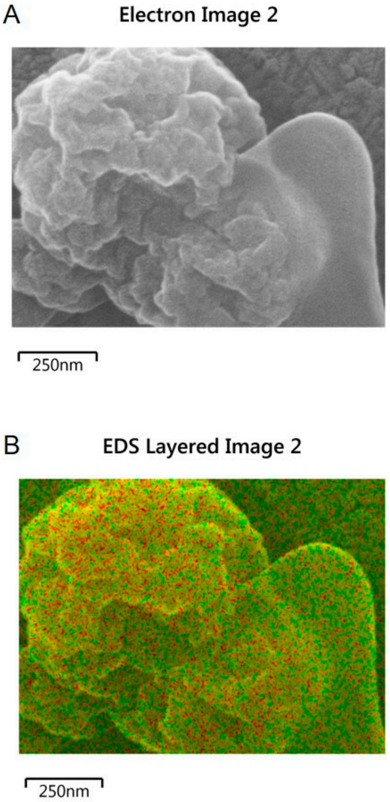

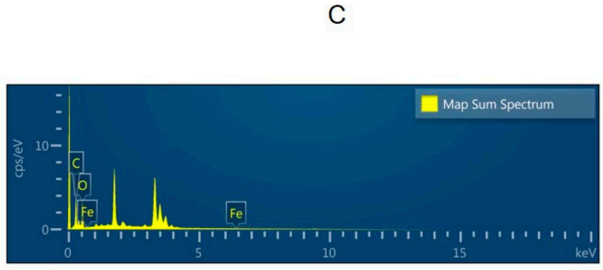

D

Element	Apparent Concentration	k Ratio	Wt%	Wt% Sigma	Atomic %
C	0.78	0.00783	47.75	0.70	54.90
O	1.49	0.00503	52.25	0.70	45.10
Fe	0.00	0.00000	0.00	0.37	0.00
Total:			100.00		100.00

Figure S1. FESEM and EDS analysis of GO.

A

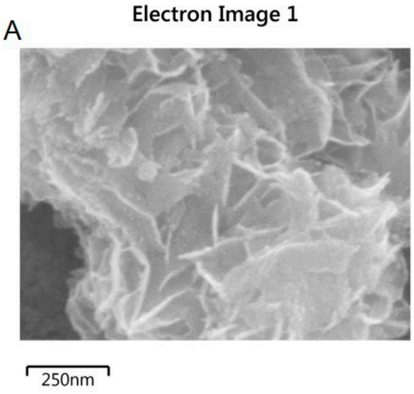

B

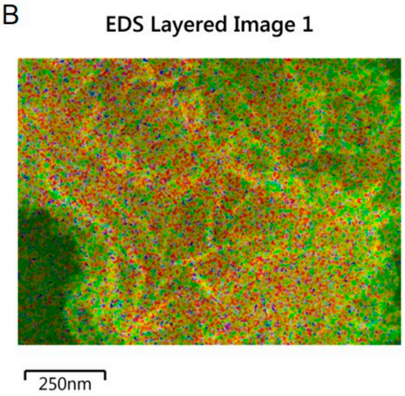

C

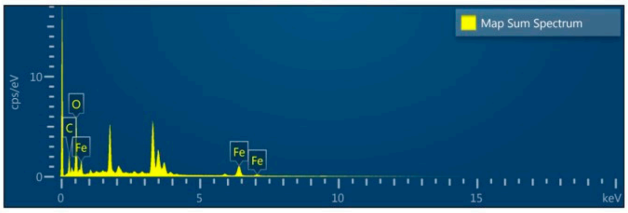

D

Element	Apparent Concentration	k Ratio	Wt%	Wt% Sigma	Atomic %
C	0.45	0.00445	29.42	0.67	42.04
O	2.64	0.00888	47.37	0.58	50.83
Fe	1.45	0.01448	23.21	0.46	7.13
Total:			100.00		100.00

Figure S2. FESEM and EDS analysis of m-rGO nanocomposite.

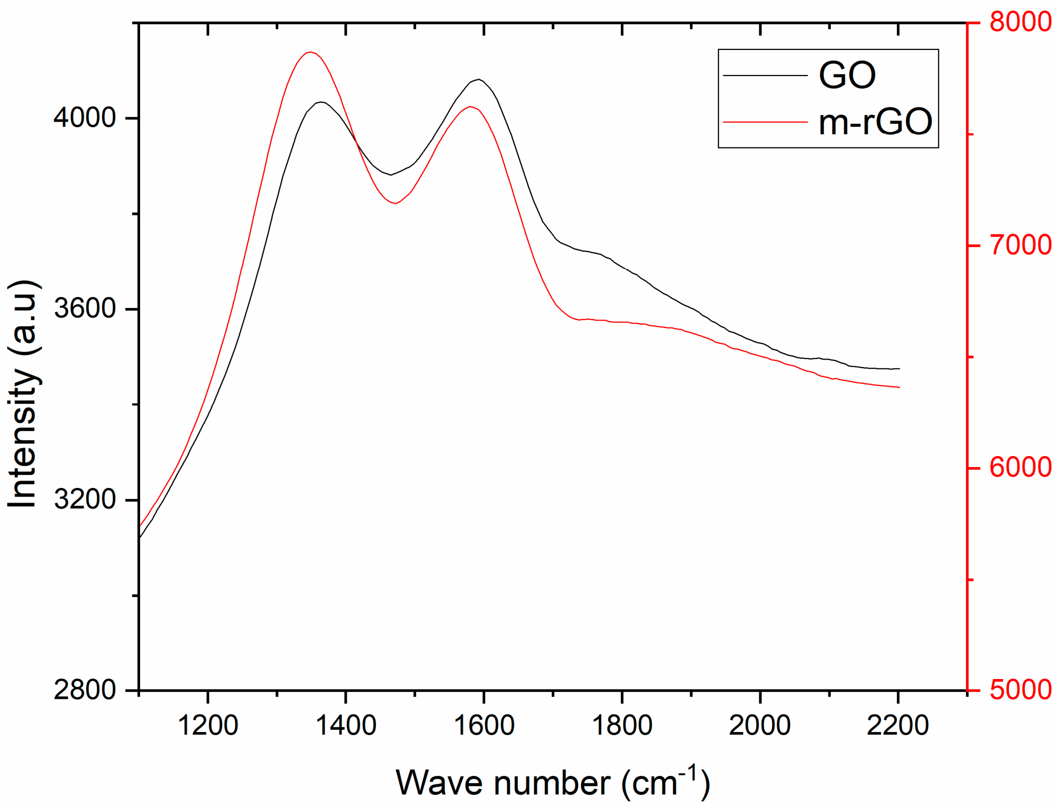

Figure S3. Raman spectra of GO and m-rGO.

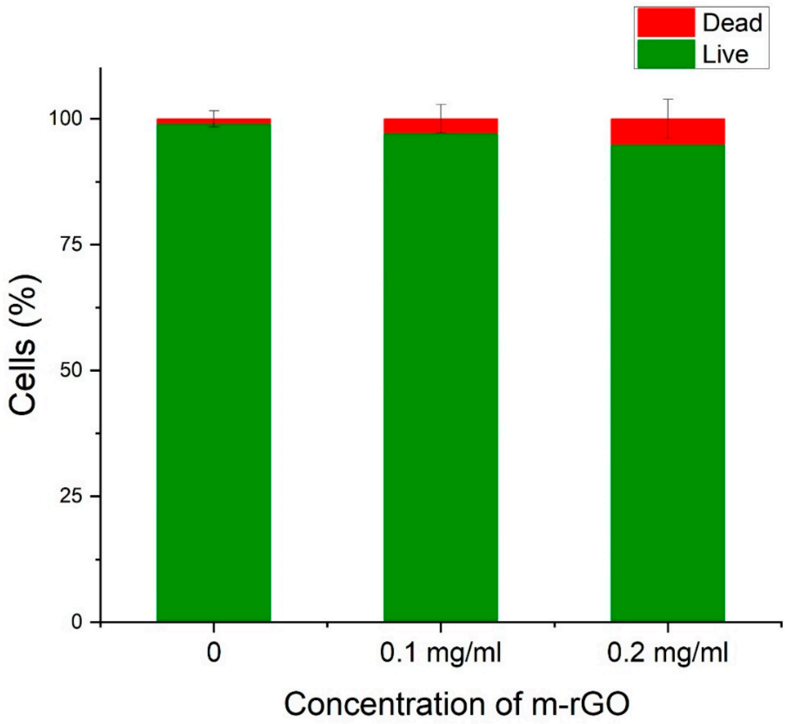

Figure S4. Quantification of Live/dead cell assay at different concentration of m-rGO.

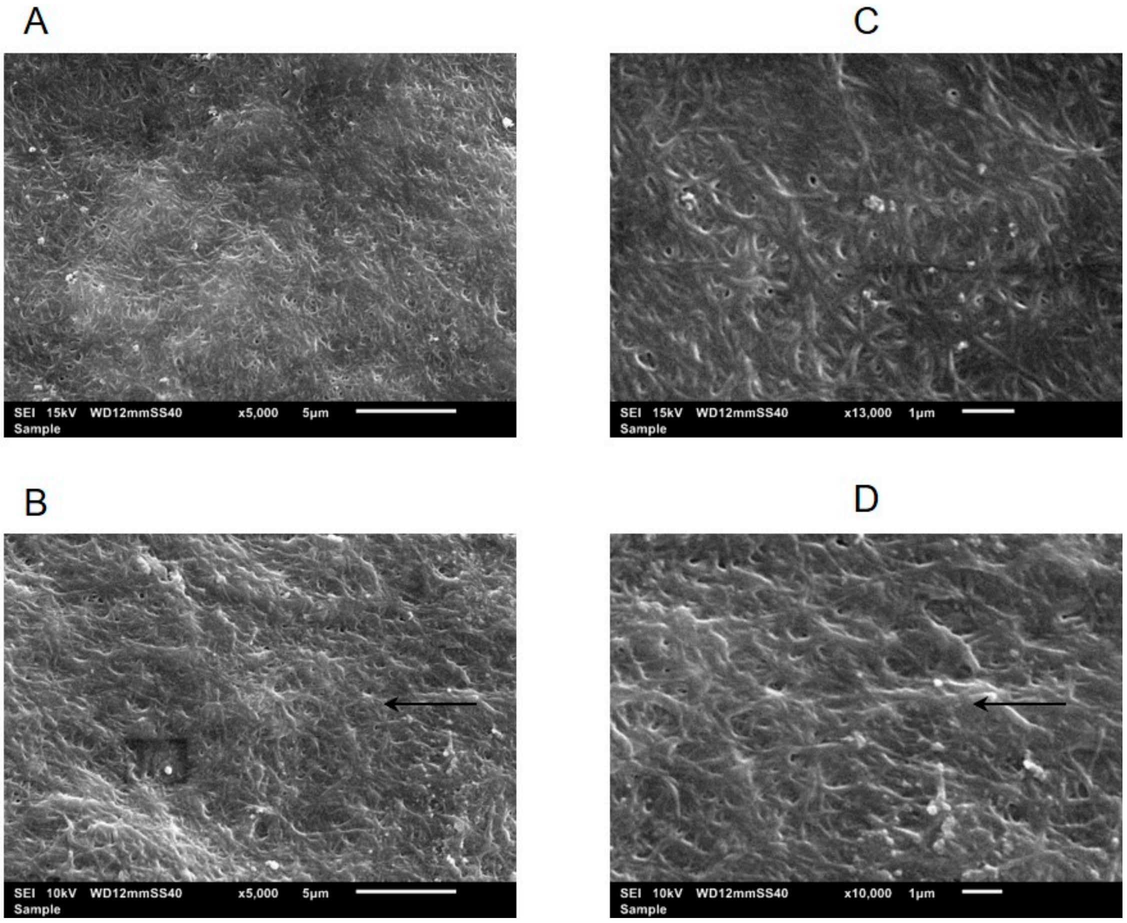

Figure S5. Scanning electron microscope images of randomly oriented (A and C) and aligned collagen fibers (B and D).

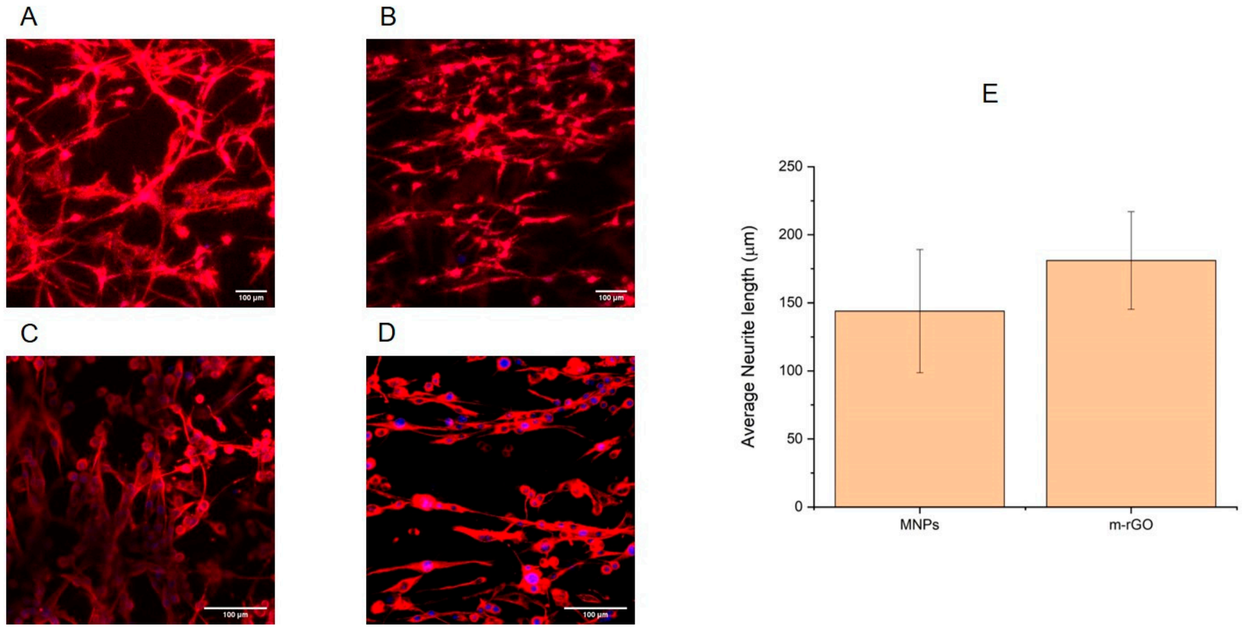

Figure S6. Cell morphology of SH-SY5Y cells within random (A and B) and aligned scaffold (C and D) of MNP-Collagen and m-rGO collagen scaffold respectively. E) Average neurite length of SH-SY5Y cells.

© 2019 by the authors. Licensee MDPI, Basel, Switzerland. This article is an open access article distributed under the terms and conditions of the Creative Commons Attribution (CC BY) license (http://creativecommons.org/licenses/by/4.0/).
